# MicroRNA-Regulated Rickettsial Invasion into Host Endothelium via Fibroblast Growth Factor 2 and Its Receptor FGFR1

**DOI:** 10.3390/cells7120240

**Published:** 2018-12-01

**Authors:** Abha Sahni, Hema P. Narra, Jignesh Patel, Sanjeev K. Sahni

**Affiliations:** Department of Pathology, University of Texas Medical Branch, 301 University Boulevard, Galveston, TX 77555-0609, USA; hpnarra@UTMB.EDU (H.P.N.); jgpatel@UTMB.EDU (J.P.)

**Keywords:** miRNA, FGF2, FGFR1, *Rickettsia*, endothelial cells

## Abstract

Microvascular endothelial cells (ECs) represent the primary target cells during human rickettsioses and respond to infection via the activation of immediate–early signaling cascades and the resultant induction of gene expression. As small noncoding RNAs dispersed throughout the genome, microRNAs (miRNAs) regulate gene expression post-transcriptionally to govern a wide range of biological processes. Based on our recent findings demonstrating the involvement of fibroblast growth factor receptor 1 (FGFR1) in facilitating rickettsial invasion into host cells and published reports suggesting miR-424 and miR-503 as regulators of FGF2/FGFR1, we measured the expression of miR-424 and miR-503 during *R. conorii* infection of human dermal microvascular endothelial cells (HMECs). Our results revealed a significant decrease in miR-424 and miR-503 expression in apparent correlation with increased expression of FGF2 and FGFR1. Considering the established phenomenon of endothelial heterogeneity and pulmonary and cerebral edema as the prominent pathogenic features of rickettsial infections, and significant pathogen burden in the lungs and brain in established mouse models of disease, we next quantified miR-424 and miR-503 expression in pulmonary and cerebral microvascular ECs. Again, *R. conorii* infection dramatically downregulated both miRNAs in these tissue-specific ECs as early as 30 min post-infection in correlation with higher FGF2/FGFR1 expression. Changes in the expression of both miRNAs and FGF2/FGFR1 were next confirmed in a mouse model of *R. conorii* infection. Furthermore, miR-424 overexpression via transfection of a mimic into host ECs reduced the expression of FGF2/FGFR1 and gave a corresponding decrease in *R. conorii* invasion, while an inhibitor of miR-424 had the expected opposite effect. Together, these findings implicate the rickettsial manipulation of host gene expression via regulatory miRNAs to ensure efficient cellular entry as the critical requirement to establish intracellular infection.

## 1. Introduction

Pathogenic *Rickettsia* species include obligate intracellular and vector-borne Gram-negative α-proteobacteria known to cause spotted fever and typhus rickettsioses in humans. As such, bacteria within the genus *Rickettsia* are divided into the spotted fever, typhus, ancestral, and transitional groups. As the respective etiologic agents of Rocky Mountain spotted fever in the Americas and Mediterranean spotted fever in the Europe and Asia, *Rickettsia rickettsii* and *R. conorii* represent two major pathogenic species belonging to the spotted fever group of rickettsiae. During infection of their mammalian hosts, rickettsiae primarily target microvascular endothelial cells (ECs) lining the small and medium-sized blood vessels, triggering host responses characterized by endothelial activation and sequelae associated with the loss of endothelial barrier integrity, leading to fluid imbalance in vital organ systems, including the skin, lungs, and brain, and thrombotic complications such as disseminated intravascular coagulation in severe cases of disease [1,2].

MicroRNAs (miRNAs) are a family of small noncoding RNAs (about 20–24 nucleotides long) capable of regulating a wide array of biological processes, including cellular development, differentiation, and proliferation; regulation of the cell cycle and metabolism; and pathways of degradation such as autophagy and apoptosis [3]. The predominant function of miRNAs is to regulate protein translation by binding to complementary sequences in the 3’ untranslated region (3’ UTR) of target messenger RNAs (mRNAs), resulting in translational repression and mRNA decay [4]. Recently, miRNAs have been shown to execute important biological roles in host–pathogen interactions, and alterations in miRNA expression are being increasingly recognized as an integral component of the host response to infection by bacterial pathogens as well as a novel molecular strategy exploited by bacteria to manipulate the mechanisms governing host defense pathways. The miRBase, one of the prominent databases of human miRNAs, currently lists more than 2500 miRNAs that have been predicted to regulate about 60% of protein-coding genes [5]. Among these, there are a number of immunologically relevant miRNAs with well-defined roles as the regulators of immune cell function and activation as well as the resolution of immune responses, establishing their contributions as important determinants of innate and adaptive immunity [6,7,8,9,10]. Furthermore, the complexity of the mechanisms underlying such fine regulation of host immune responses at the molecular level is evident in the fact that a single miRNA can interact with and bind to a number of different mRNAs, and conversely, that a single mRNA may be subjected to regulation by several miRNAs acting cooperatively to govern post-transcriptional control of mRNA translation and protein output [11]. Abundant evidence now suggests a role for host miRNAs in the replication and propagation of viruses. A generalized emerging theme in this context is the manipulation of host miRNAs to escape an antiviral response and/or to promote viral infection [12]. Similarly, virus-encoded miRNAs carrying sequences similar to or completely different than host miRNAs have also been implicated in the regulation of important biological processes, such as modulation of the viral life cycle, pathogenesis, and latency [13,14]. Along this line, a number of important bacterial pathogens, for example, *Helicobacter pylori, Listeria monocytogenes*, *Salmonella enterica* serovar Typhimurium, and *Mycobacterium tuberculosis*, among others, have also been documented to alter host miRNAs [15,16,17,18,19]. Taken together, these studies suggest that bacterial pathogens also exploit host miRNAs to ensure and prolong their survival within the host. We have recently reported on the differential expression of miRNAs and utilization of fibroblast growth factor receptor 1 (FGFR1) as one of the host cell surface receptors to facilitate entry during *R. rickettsii* and *R. conorii* infection of cultured human ECs [20,21]. In the present study, we have identified two host miRNAs that experience downregulated expression in response to rickettsial infection of human dermal, pulmonary, and cerebral ECs, in correlation with induced expression of FGF2 and FGFR1. Our results further suggest an identical pattern of alterations in the expression of these miRNAs and FGF2/FGFR1 in the lungs as a target organ system in a murine model of infection and the potential utility of these miRNAs as diagnostic biomarkers of rickettsial diseases.

## 2. Materials and Methods

### 2.1. Endothelial Cell Culture

Human dermal microvascular endothelial cells (HMECs) were obtained from the Centers for Disease Control and Prevention (Atlanta, GA). HMECs were cultured in MCDB131 medium (Caisson’s Laboratories) supplemented with Fetal Bovine Serum (FBS) (10% *v*/*v*; Aleken Biologicals), epidermal growth factor (10 ng/mL, Thermo Fisher Scientific, Waltham, MA, USA), L-glutamine (10 mM, Thermo Fisher Scientific), and hydrocortisone (1 µg/mL, Sigma) [22]. Human cerebral microvascular endothelial cells (HCECs) were kindly provided by R. K. Yu and S. S. Dasgupta, Institute of Molecular Medicine and Genetics, Medical College of Georgia, Augusta, GA. These immortalized cell lines, which display typical morphological, phenotypic, and functional characteristics of microvascular endothelium, were grown in culture as recommended [23,24]. Primary human lung microvascular ECs (HLMECs) were purchased from Lonza and maintained in culture according to the manufacturer’s instructions. All cell cultures were incubated and maintained at 37 °C in an incubator with 5% CO_2_.

### 2.2. Cell Infection and Transfection

*R. conorii* (strain Malish 7) and *R. rickettsii* (Sheila Smith) were grown in cultured Vero cells, purified by differential centrifugation as described previously [25], and the stocks were aliquoted as volumes of ≤500 µL and kept frozen at −80 °C to avoid freeze–thaw cycles. The infectivity titers of purified stocks were estimated by citrate synthase (gltA)-based quantitative PCR and plaque formation [26]. ECs were seeded at a dilution to achieve 80% to 90% confluence and infected with approximately 6 × 10^4^ plaque forming units (pfu) for every cm^2^ of culture surface area with *R. conorii* or *R. rickettsii* to achieve approximately 5 intracellular rickettsiae per cell according to our standard established procedures [20,22]. At different times post-infection, culture medium was removed by gentle aspiration and the cells were directly lysed in TRI Reagent^®^ (Molecular Research Center). In all experiments, the viability of both mock controls and *Rickettsia*-infected ECs was ascertained microscopically.

The mimics and inhibitors for miR-424 and miR-503, along with the negative controls (mirVana™ miRNA mimic and inhibitor negative controls), were purchased from Applied Biosystems/Thermo Fisher Scientific. The miRNA mimics were transfected for 24 h, while the miRNA inhibitors were transfected into ECs for 72 h using Lipofectamine RNAiMAX according to the manufacturer’s recommendations prior to infection with *R. conorii* for 6 h.

### 2.3. RNA Preparation

Total RNA was extracted from *R. conorii*-infected and corresponding mock control ECs according to our standard TRI Reagent^®^ protocol optimized in accordance with the manufacturer’s recommendations as described previously [21]. For RNA isolation from blood, the MagMAX mirVana Total RNA Isolation kit (Thermo Fisher Scientific) was used. The resultant RNA preparations were subjected to treatment with DNase I to remove contaminating genomic DNA and quantified using a MultiSkan™ Go Spectrophotometer (Thermo Scientific). The RNA quality was then assessed by visualization of 18S and 28S RNA bands on an Agilent Bioanalyzer 2100 (Agilent Technologies, Santa Clara, CA, USA). The electropherogram for each sample was used to determine the 28S:18S ratio and the RNA integrity number (RIN) [27]. RNA preparations with a RIN number ≥9.0 were used in further experiments.

### 2.4. Quantitative Real-Time PCR

TaqMan^®^ two-step RT-PCR assays containing primers for both miRNA-specific reverse transcription and quantitative PCR were obtained from Applied Biosystems. Total RNA (1 µg) for each sample was reverse-transcribed using the TaqMan MicroRNA cDNA synthesis kit (Applied Biosystems) and miRNA-specific primers for miR-424 and miR-503 as well as oligo (dT) primers for concurrent analysis of 18S (18S ribosomal RNA) and Glyceraldehyde 3-phosphate dehydrogenase (GAPDH) expression as a house keeping control. The expression of miRNAs was analyzed by real-time PCR using the TaqMan^®^ assay specific for each microRNA (Applied Biosystems). 18S RNA was employed as an endogenous control and used to normalize for miRNA expression. The mRNA expression of FGF2 and FGFR1 was measured using the gene-specific TaqMan primers, and GAPDH was utilized as an endogenous control to normalize the mRNA expression between different samples [20]. The ^Δ^Ct values for experimental (infected) samples were compared to the baseline mock control cells, which were assigned a value of 1, and the relative expression was determined by comparative Ct (^ΔΔ^CT method) as described earlier [20]. Briefly, we measured the amplification of the target and housekeeping genes in infected and control samples, and the Ct values for the target genes were normalized to the housekeeping species using the StepOne™ Plus software version 2.3. We next determined the relative quantitation by comparing normalized target quantity in each experimental (infected) sample to the normalized target quantity in mock controls (uninfected). For determining the rickettsial copy number, total DNA (host and rickettsial) was extracted using the DNeasy Blood and Tissue Kit (Qiagen, Germantown, MD, USA) according to the manufacturer’s instructions and quantified by a spectrophotometer (Thermo Fisher Scientific). Quantitative PCR was performed using the rickettsial outer membrane protein A (ompA) primer pair RR190.547F and RR190.701R for spotted fever group rickettsiae [28].

### 2.5. In Vivo Model of Infection

All animal experiments were performed in accordance with the research protocol approved by the Institutional Animal Care and Use Committee. The University has a file with the Office of Laboratory Animal Welfare and an approved Assurance Statement (#A3314-01). C3H/HeN mice (Charles River) were infected with 2.25 × 10^5^ pfu of *R. conorii* per animal administered intravenously (IV). The control animals received an IV injection of the identical volume of saline. Four animals were used per group in two independent experiments, i.e., *n* = 8. On day 3 post-infection, mice were anesthetized by inhalational isofluorane for collection of blood by cardiac puncture, after which the mice were euthanized and the lungs were removed aseptically and preserved in an RNAlater solution for isolation of total RNA and analysis by q-RT-PCR using miRNA-specific Taqman assays (Applied Biosystems, Waltham, MA, USA).

### 2.6. Statistical Analysis

All experiments were performed at least three times with technical triplicates to calculate the results as the mean ± standard error (SE). Statistical analysis for differentially expressed miRNAs in *R. conorii*-infected and mock control groups was performed by one/two-way ANOVA with Dunnett’s post-test using GraphPad Prism 4.00. The *p* value for statistical significance among experimental conditions being compared was set at ≤0.05.

## 3. Results 

**Figure 1:**
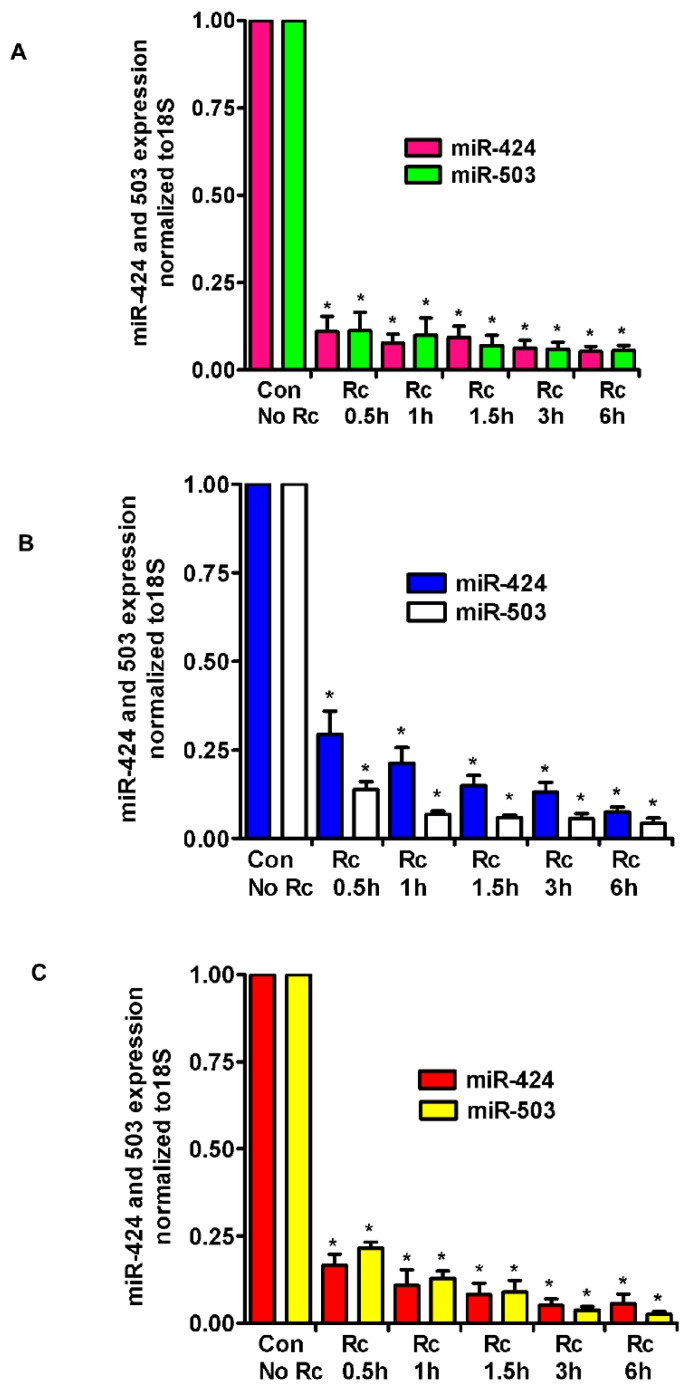
Expression levels of miR-424 and miR-503 in different types of endothelial cells infected with *R. conorii*. HMECs (**A**), HLMECs (**B**), and HCECs (**C**) were infected with *R. conorii* (Rc) for various time periods up to 6 h. RNA was extracted and qRT-PCR assays were performed to measure the expression of miR-424 and miR-503. The data was normalized to 18S RNA, and relative expression was calculated by the ^ΔΔ^Ct method. The results are presented as the mean ± standard error (SE) of three independent experiments. The asterisk indicates statistically significant change (*p* ≤ 0.01). HMECs: human dermal microvascular endothelial cells; HLMECs: human lung microvascular endothelial cells; HCECs: human cerebral microvascular endothelial cells; Con: control.

A recently published study and miRNA databases report fibroblast growth factor (FGF2) and its receptor (FGFR1) as validated targets for miR-424 and miR-503 [29]. Also, we have recently demonstrated the involvement of fibroblast growth factor receptor 1 (FGFR1) in the internalization of *R. conorii* into host endothelium in vitro and during *R. conorii* infection in vivo [21]. Therefore, we investigated the levels of expression of miR-424 and miR-503 in *R. conorii*-infected human ECs. The tropism for the vascular endothelium lining of small and medium-sized vessels in vivo is intriguing, considering that pathogenic rickettsiae are capable of infecting a wide range of cultured cell types in vitro. During natural infections, rickettsiae enter through the skin, primarily affecting the lungs and the brain and causing pulmonary and cerebral edema due to compromised vascular permeability. We, therefore, chose to study all three types, i.e., dermal (HMECs), cerebral (HCECs), and lung (HLMECs) ECs, to measure the expression of miR-424 and miR-503. Confluent ECs were infected with *R. conorii* for various times (0.5, 1, 1.5, 3, and 6 h), total RNA was isolated, and miR-424/503 expression was measured by qRT-PCR using Taqman miR-specific primers. As compared to the baseline in mock control HMECs, there was a dramatic decrease in the expression of miR-424 and miR-503 as early as 30 min post-infection (83.3 ± 3% and 78.4 ± 4%, respectively, *p* ≤ 0.01, Figure 1A). Significant and nearly identical downregulation of the expression of both miRNAs were observed up to 6 h post-infection as compared to the mock controls. In the lung microvascular ECs (HLMECs), expression of miR-424 and miR-503 was also significantly downregulated (86.9 ± 5% and 94.4 ± 3% at 3 h and 92.6 ± 3% and 95.7 ± 3% at 6 h, respectively, *p* ≤ 0.01) in infected cells as compared to the mock controls (Figure 1B). Similar results were obtained in the cerebral microvascular ECs (HCECs), where the expression of miR-424/503 in infected cells was downregulated by 94.8 ± 3% and 96.3 ± 2% at 3 h and 94.3 ± 5% and 97.4 ± 2% at 6 h post-infection, respectively (*p* ≤ 0.01) (Figure 1C). Overall, all three types of ECs demonstrated a similar pattern of dramatic downregulation of both miRNAs in infected cells. In addition, HMECs infected with *R. rickettsii,* another spotted fever group *Rickettsia* species, also displayed a similar pattern of regulation of expression for both miRNAs, where miR-424 depicted 96 ± 6% and miR-503 showed 93 ± 4% downregulation 6 h post-infection as compared to the mock controls. 

**Figure 2:**
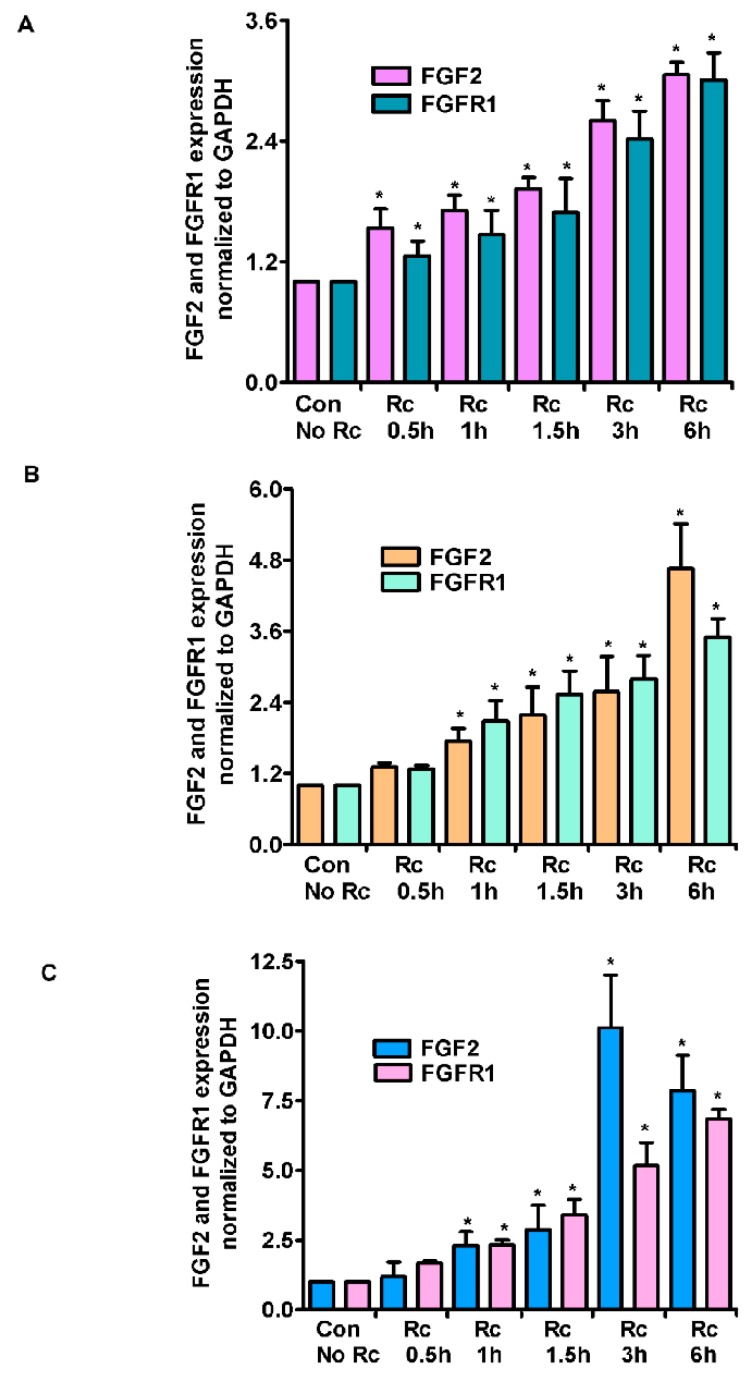
Expression levels of FGF2 and FGFR1 mRNA in *R. conorii*-infected host endothelial cells. HMECs (**A**), HLMECs (**B**), and HCECs (**C**) were infected with *R. conorii* for various time periods up to 6 h. RNA was extracted, and qRT-PCR assays were performed to measure the FGF2 and FGFR1 expression. The data was normalized to 18S rRNA and relative expression was calculated by the ^ΔΔ^Ct method. The results are presented as the mean ± SE of three independent experiments. The asterisk indicates statistically significant change (*p* ≤ 0.01).

As a follow up to changes in miR-424 and miR-503 expression, we next measured the expression of FGF2 and its receptor FGFR1 in *R. conorii*-infected ECs. In HMECs, we observed a time-dependent increase in the expression levels of both FGF2 and FGFR1, with the maximum increase at 6 h post-infection (3.06 ± 0.2- and 3.01 ± 0.5-fold, respectively, *p* ≤ 0.01) (Figure 2A). In HLMECs, there was a similar time-dependent increase in the steady-state mRNA levels for both FGF2 and FGFR1, as evidenced by a 4.65 ± 0.7- and 3.5 ± 0.4- fold increase (*p* ≤ 0.01) in their expression, respectively (Figure 2B). Lastly, HCECs also displayed a similar, but much larger increase in the mRNA expression levels of both FGF2 and FGFR1 at 6 h post-infection, when the infected cells exhibited about 7.87 ± 1.0-fold increase in FGF2 and 6.83 ± 0.6-fold increase in FGFR1 mRNA levels as compared to the mock controls (*p* ≤ 0.01) (Figure 2C). A plausible explanation for only a modest increase in the expression of FGF2/FGFR1 levels in HMECs is that these cells display comparatively higher basal expression of FGF2 and FGFR1. Again, *R. rickettsii* infection also resulted in a similar pattern of induced FGF2/FGFR1 mRNA expression in HMECs, where we observed a 3.21 ± 0.6-fold increase in FGF2 expression and a 2.84 ± 0.2-fold increase in FGFR1 expression in infected cells compared to the mock controls.

**Figure 3:**
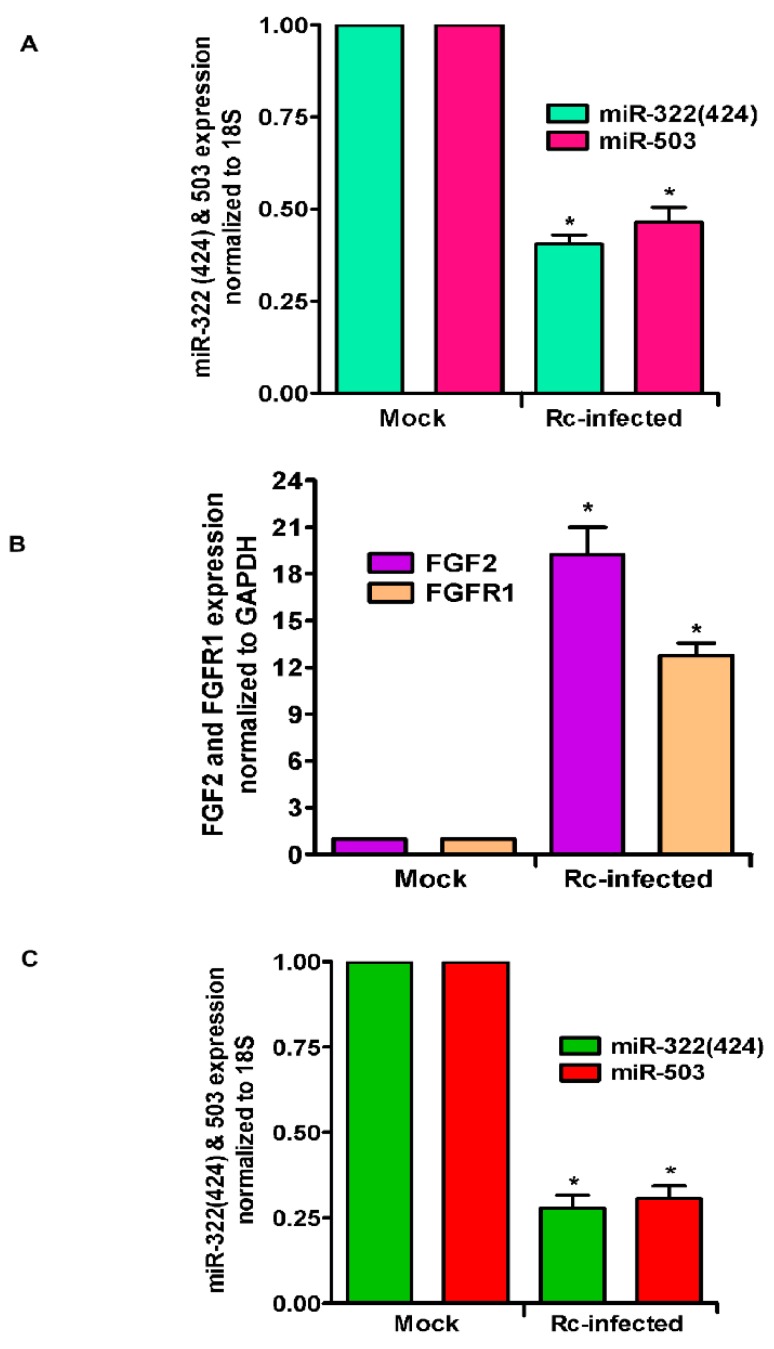
Expression of miR-424(322), miR-503, and FGF2/FGFR1 in *R. conorii*-infected C3H/HeN mice in vivo. Mice were infected with *R. conorii* (2.25 × 10^5^ pfu) intravenously (IV). Control animals received IV injection of saline. On day 3 post-infection, mice were anesthetized, blood and lungs were collected, RNA was isolated, and expression of both miRNAs in the lungs (**A**) and blood (**C**) and FGF2/FGFR1 in the lungs (**B**) was measured by qRT-PCR. The asterisks indicate statistically significant change (*p* ≤ 0.01).

As an important corollary to in vitro findings, we further analyzed the expression level of miR-322 (mouse orthologue of miR-424) and miR-503 in the lungs of mice infected with *R. conorii*. qRT-PCR revealed about 61.5 ± 4.4% and 54.5 ± 6.8% reduction in the levels of miR-322 and miR-503, respectively, on day 3 post-infection as compared to the lungs of mock control mice (*p* ≤ 0.01 for both) (Figure 3A). Subsequent determination of FGF2 and FGFR1 mRNA levels demonstrated an 18.2 ± 4.4-fold increase in the steady-state expression of FGF2 and a 12.3 ± 2.3-fold increase in FGFR1 in the lungs of infected mice in direct comparison to their basal expression in the lungs of mock control animals (Figure 3B). Since miRNAs are also increasingly recognized for the potential to be used as biomarkers in various disease conditions including infection, we next determined the levels of these two miRNAs in the blood of mice infected with *R. conorii*. Our findings clearly demonstrate about a 72.1 ± 5% and 69.4 ± 5% reduction in the expression of both miR-322 and miR-503, respectively, in the blood of infected mice, representing a significant change (*p* ≤ 0.01) in comparison to their basal levels in a corresponding cohort of mock control animals (Figure 3C). Together, these findings recapitulate in vitro changes observed in cultured ECs for both miRNAs and expression levels of FGF2 and FGFR1 in an established mouse model of rickettsial infection. 

**Figure 4:**
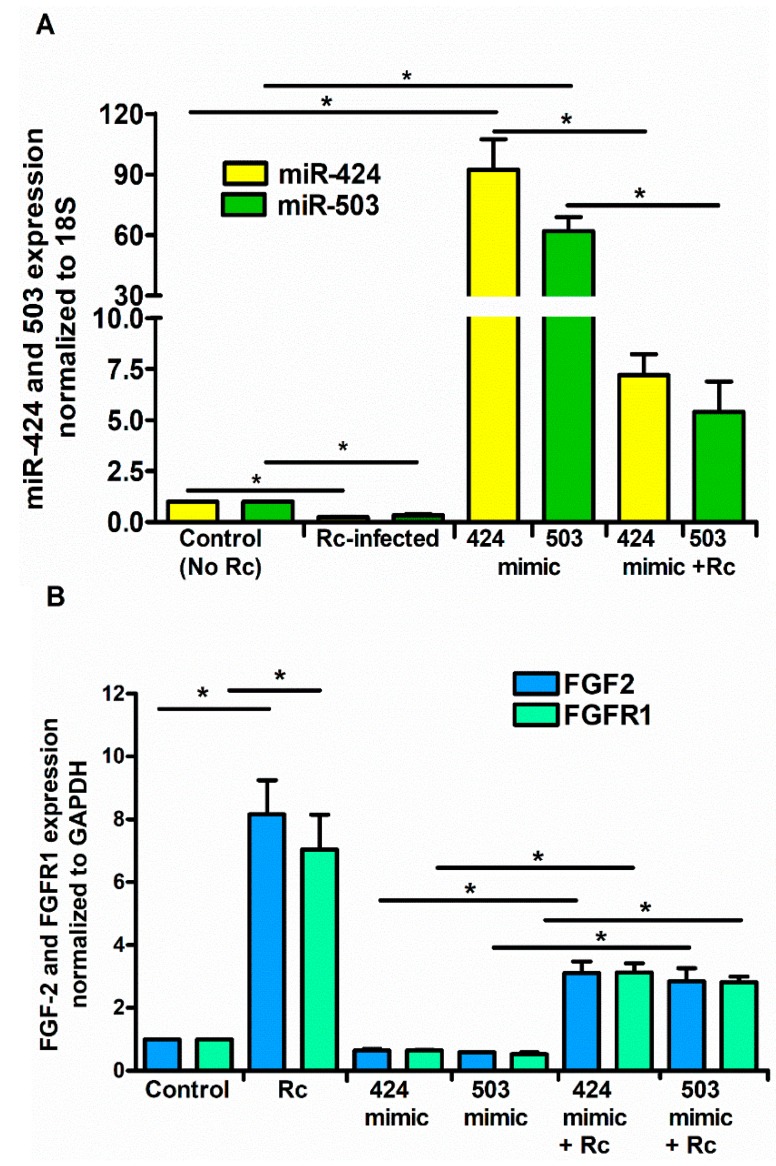
Effect of miRNA mimics on the expression levels of FGF2/FGFR1. ECs were transfected with miR-424 or miR-503 mimic (1 nM) for 24 h using Lipofectamine RNAiMAX according to the manufacturer’s instructions prior to infection with *R. conorii* for 6 h. Cells were lysed in Tri Reagent for isolation of total RNA. The expression of miR-424 and miR-503 (**A**) and FGF2/FGFR1 (**B**) was measured by qRT-PCR. The data are presented as the mean ± SE of three independent experiments, and the asterisk (* *p* ≤ 0.01) indicates statistically significant change.

To ascertain whether or not miR-424 and miR-503 function as the regulators of FGF2/FGFR1 mRNA during *R. conorii* infection, we next performed a series of gain- and loss-of-function experiments using miRNA-specific mimics or inhibitors. We conducted these studies using HCECs, based on highly significant changes in both miRNAs and FGF2/FGFR1 expression in this particular cell type in response to rickettsial infection. To this end, miRNA-mimics and inhibitor sequences specifically targeting miR-424 and miR-503, along with a negative control (mirVana™ miRNA mimic negative control), were transfected into HCECs using Lipofectamine^®^ RNAiMAX, and their effects on miR-424, miR-503, and FGF2/FGFR1 expression were determined by qRT-PCR. As expected, introduction of the mimics resulted in a dramatic increase in miR-424 and miR-503 expression. Interestingly, infection with *R. conorii* was able to counteract the effects of miR-mimics, resulting in reduced miR-424 and miR-503 expression in HCECs transfected with the mimic when compared to the corresponding mock controls (Figure 4A). Conversely, mRNA expression of FGF2/FGFR1 was significantly downregulated in cells transfected with miR-424 and miR-503 mimics alone and those infected with *R. conorii* following the delivery of mimics for both miRNAs.

**Figure 5:**
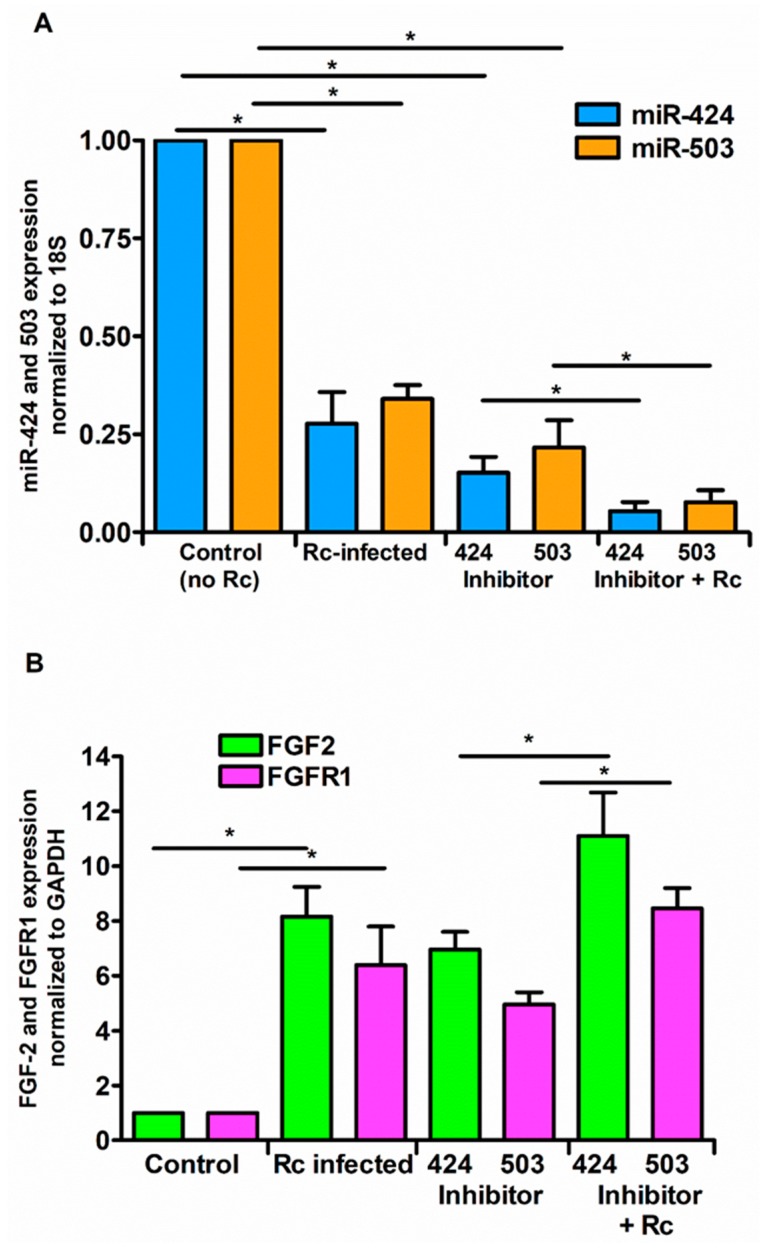
Effect of miRNA inhibitors on expression levels of FGF2/FGFR1. ECs were transfected with miR-424 or miR-503 inhibitor (200 nM) for 72 h using Lipofectamine RNAiMAX according to the manufacturer’s instructions and then infected with *R. conorii* for 6 h. Cells were lysed in Tri Reagent and total RNA was isolated for measuring the expression of miR-424 and miR-503 (**A**) and FGF2/FGFR1 (**B)** by qRT-PCR. The data are presented as the mean ± SE of three separate experiments. The asterisk (* *p* ≤ 0.01) indicates statistically significant change.

In contrast to our findings with the mimics, the miR-424 and miR-503 inhibitors reduced the cellular miRNA levels by about 75%, while *R. conorii* infection further reduced the miR-424 and miR-503 expression by about 50% in the presence of the inhibitors specific to these miRNAs (Figure 5A). Accordingly, opposite effects on FGF2/FGFR1 mRNA levels were also clearly evident when the inhibitors of miR-424 and miR-503 were used in these experiments (Figure 5B). Together, these findings yield evidence for the direct involvement of miR-424 and miR-503 in the regulation of FGF2/FGFR1 expression during rickettsial infection of host ECs.

**Figure 6:**
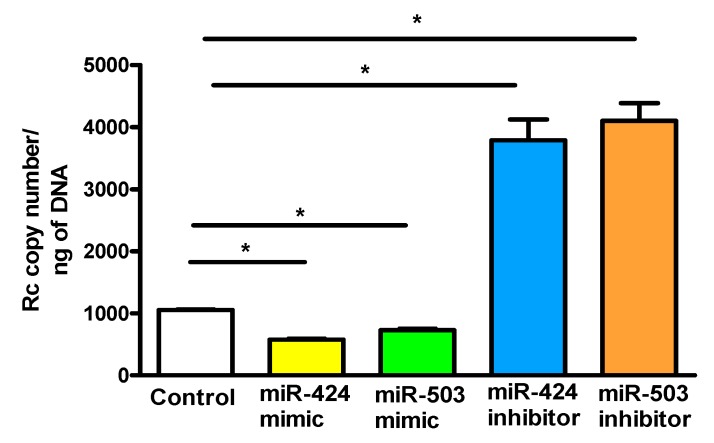
miRNA-mediated rickettsial internalization into host endothelial cells: ECs were transfected with a miR-424 mimic (1 nM) or inhibitor (200 nM) prior to infection with *R. conorii* for 6 h. Cells were lysed and DNA was isolated using a Qiagen kit for determination of the copy number of rickettsiae by qPCR. The data are presented as the mean ± SE of three separate experiments. The asterisk (* *p* ≤ 0.01) indicates statistically significant change.

Based on our recent findings implicating FGFR1 in rickettsial internalization into ECs and evidence for miR-424 and miR-503-mediated regulation of FGF2/FGFR1 expression, we next investigated the effects of miR-424 and miR-503 mimics and inhibitors on *R. conorii* internalization into ECs. ECs transfected with the mimics or inhibitors of miR-424 and miR-503 were infected with *R. conorii* and the copy number of internalized rickettsiae was determined (Figure 6). Our results suggest that miR-424 and miR-503 mimics significantly inhibit *R. conorii* internalization, whereas inhibitors of both miRNAs have an opposite enhancing effect of facilitating rickettsial entry into ECs. These results corroborate our earlier findings that FGF2/FGFR1-mediated entry of *R. conorii* into host ECs is regulated by miR-424 and miR-503.

## 4. Discussion 

MicroRNAs play a major role in human diseases, with the aberrant expression of miRNAs capable of interacting with several oncogenes and tumor suppressors now reported in all cancers [30]. In addition, miRNA regulatory networks comprised of either a single miRNA or those executing their effects as clusters consisting of either related family members or disparate miRNAs can impact the mechanisms responsible for normal physiology as well as nonmalignant disorders [31]. Rapidly accumulating evidence implies important roles for miRNAs in the modulation of inflammatory responses, cell penetration, innate and adaptive immunity, and tissue remodeling consequent to infection as critical attributes of intricate and complex interactions between bacterial pathogens and their hosts. For example, miR-146 and miR-155 represent two well-studied miRNAs, due in large part to their roles in the regulation of inflammation and immunity during bacterial infections [17]. Yet, another emerging theme is the possibility of their exploitation as circulating biomarkers for bacterial infections (for example, pulmonary tuberculosis and *H. pylori*-associated gastritis) and the potential for their application as novel therapeutic targets [32,33].

Pathogenic *Rickettsia* species are known to target the microvascular endothelial lining of small and medium-sized blood vessels during human infections and to exploit redundant mechanisms to gain entry, for release into the cytoplasm as free intracytoplasmic energy parasites, and for survival inside the host cell [1,2]. As a ubiquitous and multifunctional regulator of the proliferation, differentiation, and angiogenic potential of different mammalian cells, FGF2 is an important protein belonging to the large family of fibroblast growth factors. It is involved in both morphogenic and mitogenic pathways and regulates a variety of important cellular functions underlying developmental processes by binding to and activating the receptor tyrosine kinases FGFR1–FGFR4 [34]. A fifth receptor, FGFR5 (also known as FGFRL1) can also bind FGF2, but is devoid of a tyrosine kinase domain and may negatively regulate signaling [35]. Derived from a single mRNA, there are five different isoforms of FGF2 (34, 24, 22.5, 22, and 18 kDa), of which the four high-molecular-weight forms arise from the upstream CUG codons, whereas the 18 kDa isoform arises from the downstream AUG codon [36,37]. The human FGFRs, FGFR1 through 4, are a subfamily of receptor tyrosine kinases associated with the activation of multiple cell signaling cascades and responses such as proliferation, differentiation, and survival. Although all of these FGFRs are expressed on various cells and tissues at varying levels and FGF2 interacts with them all, FGFR1 has the highest affinity for FGF2 and is primarily responsible for FGF2-induced signaling in ECs, which determines multiple vascular endothelial functions, including growth, migration, and angiogenesis [38,39,40].

Published reports have implicated miR-424 and miR-503 in the regulation of expression of FGF2 and FGFR1 in ECs [29,41], and our laboratory has recently identified FGFR1 as one of the host cell receptors exploited by spotted fever rickettsiae for internalization into host ECs [21]. These findings served as the rationale for further investigation of the expression of miR-424/503 in human dermal, pulmonary, and cerebral microvascular ECs during *R. conorii* infection in vitro [42]. Our results for this aspect of the study convincingly demonstrate a dramatic downregulation of both miRNAs and significantly increased mRNA expression of FGF2/FGFR1 in infected ECs over the basal levels of expression in mock controls. Because miR-424 (miR-322 in rodents) and miR-503 are co-transcribed as a polycistronic primary transcript (pri-miRNA) and comprise the miR-424(322)/503 cluster [43,44], it is not surprising that both miRNAs exhibit an identical pattern of transcriptional regulation. In addition, both miR-424 and miR-503 regulate the expression of FGF2 and FGFR1 by binding to the 3′-UTR sequences [41,45] and FGF2 upregulates the expression levels of mature miR-424, clearly establishing a regulatory loop between miR-424 and FGF2 [29]. Importantly, as a follow-up to our in vitro findings, we have further ascertained the decreased expression of both miRNAs and increased expression of FGF2/FGFR1 mRNAs in the lungs as one of the major target organs to illustrate that altered miRNA/mRNA levels in three different types of cultured ECs potentially correlate with in vivo changes in an established murine model of spotted fever rickettsiosis. The *R. conorii* mouse model of infection has routinely been used for in vivo investigations because *R. conorii* infection in susceptible C3H/HeN mice closely mimics the disseminated endothelial infection, which is the major feature of pathogenesis and displays the overall pathology of Rocky Mountain Spotted Fever (RMSF) and Mediterranean Spotted Fever (MSF) in humans. Also, a direct and authenticated *R. rickettsii* mouse model is not yet available, mainly due to resistance of a number of mouse strains to pathogenic *R. rickettsii* [46,47].

Innate immunity is the first line of defense in response to invading pathogens, and the importance of miRNAs as determinants of host–pathogen interactions now represents a rapidly emerging area of enquiry, due mainly to the importance of miRNAs in the modulation of the host cell transcriptome and host immune responses towards microorganisms. In ECs, several miRNAs have been shown to be involved in the control of a variety of physiological and pathological functions, including angiogenesis, regulation of oxidative stress and antioxidant mechanisms, nitric oxide release, vascular inflammation, and mediation of intercellular communication [42]. As a single layer of cells lining the entire vascular tree throughout the body, the microvascular endothelium plays an important role in the regulation of hemostasis and functions as a transport gatekeeper for the exchange of substances such as nutrients, hormones, and metabolic waste. It is also important to consider, however, that microvascular ECs in the capillary beds of different tissues are endowed with distinct structural, phenotypic, and functional attributes. Accordingly, organ-specific ECs have distinct expression patterns of gene clusters to support functions that are unique and critical to the development of that particular organ system and tend to display distinct barrier properties, angiogenic capabilities, and metabolic profiles. Because pulmonary and cerebral edema are prominent pathologic features of human rickettsial infections, suggesting critical involvement of the microvasculature of the lungs and brain in disease pathogenesis, we compared the expression levels of miR-424 and miR-503 in dermal, brain, and cerebral ECs. Interestingly, our findings reveal a similar pattern of significant downregulation for both miRNAs and concordant increase in FGF2/FGFR1 expression in these ECs, with the most striking changes in cerebral ECs. These results are in general agreement with our previous findings that both macro- as well as microvascular ECs infected in vitro with spotted fever rickettsiae display relatively similar responses in regard to the activation of signal transduction cascades, expression and secretion of cytokines and chemokines, and induction of oxidative stress and consequent antioxidant mechanisms [22].

FGF2 and FGFR1 are primary regulators of ECs proliferation and angiogenesis, and FGF2 exerts its proangiogenic effects via the activation of FGFR1. Therefore, it is possible that in addition to facilitating the process of pathogen internalization, miRNA-governed enhancement of FGF2 and FGFR1 expression may promote endothelial proliferation, providing the host cellular niche critical for the survival, growth, and replication of pathogenic rickettsiae, being intracellular parasites. In addition, FGF2 also resides in the extracellular matrix (ECM), where it is tightly bound to heparan sulfate proteoglycans, which protects it from proteolysis and limits its diffusion through the extracellular matrix to potentiate its regulatory effects via signaling through FGFRs [48,49]. Also, a variety of microbes interact with different ECM proteins to effectively establish an infection, evade immune responses, and spread from cell to cell [50]. Since the ECM is intimately involved in cell adhesion and cell-to-cell communication, FGF2 may also play a role in facilitating the intercellular spread of rickettsiae. In addition, truncated forms of FGFR1 have been demonstrated to freely circulate in the blood [51], lending support to the possibility of its involvement in the systemic dissemination of rickettsiae during infection of the mammalian hosts. 

The miRNA profiling for markers of human diseases has been performed with success in biological samples such as cerebrospinal fluid, peripheral blood cells, plasma, serum, and whole blood. The discovery of circulating miRNAs in peripheral blood and the evidence for their stability in the blood has led to the completion of several investigations confirming the differential expression of specific miRNAs and their potential use as diagnostic and prognostic markers of human disease. The findings of this study reveal substantial downregulation of miR-322/503 expression in the serum of infected mice as compared to the corresponding control subjects, suggesting the usefulness of these two miRNA candidates as potential biomarkers of human rickettsial infections. In summary, the present study illustrates that miR-424/503 are significantly downregulated in three different types of ECs representing the primary targets of infection in humans, and that such downregulation may promote high levels of FGFR1 expression to facilitate subsequent pathogen invasion and/or dissemination. Further functional analysis to determine the precise roles of miR-424 and miR-503 in the host–pathogen interplay and pathophysiology should lead to the development of novel diagnostics and/or therapeutics to combat the scourge of human spotted fever rickettsioses.
